# Exploring the dynamics of allostery through multi-dimensional crystallography

**DOI:** 10.1007/s12551-024-01224-3

**Published:** 2024-09-19

**Authors:** C. E. Hatton, P. Mehrabi

**Affiliations:** 1 Institute for Nanostructure and Solid-State Physics, University of Hamburg, Hamburg, Germany; 2 Max-Planck-Institute for the Structure and Dynamics of Matter, Hamburg, Germany

**Keywords:** Time-resolved crystallography, Temperature, Allostery, Enzymes

## Abstract

By delving into the applications, methodologies, and case studies of multi-dimensional crystallography, whereby time and temperature are varied in the context of allostery, this review aims to elucidate the intricate interplay between structure, dynamics, and function in allosteric proteins. As the field of structural biology continues to advance, integrating multi-dimensional approaches promises to unlock new frontiers in our quest to decipher the molecular mechanisms governing life.

## Introduction

At the beginning of protein crystallography in the 1950s, structure solutions of the first heme-proteins marked a pivotal moment for structural biology by revealing how changes in protein conformation are linked to protein regulation in the form of allostery—a concept that has significantly shaped our understanding of protein regulation (Wyman 1948; Monod et al. 1965; Koshland et al. 1966). Allosteric regulation was originally viewed as a static, two-state model solely regulated by ligand binding. In the early 1960s, two models were proposed, the Monod–Wyman–Changeux (MWC) and the Koshland, Némethy, and Filmer (KNF) model (Monod et al. 1965; Koshland et al. 1966). Both depicted the protein in either an active or inactive conformation. The primary difference was in how the changes in conformation occurred. While the MWC model proposed that the conformational changes that take place occurred in a concerted fashion for the different subunits in the protein, the KNF model proposed that the changes occurred sequentially (Monod et al. 1965; Koshland et al. 1966). The KNF model also proposed the idea of negative cooperativity, where ligand binding would cause a reduction in the affinity of the other binding sites (Monod et al. 1965; Koshland et al. 1966).

Early crystallographers faced substantial challenges, with the difficulty of obtaining structures and the limited resolution of early static structures. Nevertheless, they were very proficient at catching intermediate states—as can be seen in early trapping literature(Blake et al. 1967; Henderson 1970; Makinen and Fink 1977; James 1980) —these limitations underscored the need to be creative with their experimental designs. A provocative take is that the rapid advance and adoption of cryo-crystallography by the broader protein community likely slowed down the development of time-resolved crystallography. Advances in the field of crystallography have furthered our understanding of these dynamic processes. Likewise, the development of time-resolved techniques has enabled observations of these changes in real time, while structures obtained at ultra-high resolutions enable highly detailed modelling of the subtle structural changes that underpin many allosteric processes (Ren et al. 1999; Srajer and Royer 2008; Šrajer and Schmidt 2017; Chapman 2019; Pearson and Mehrabi 2020; Nass et al. 2020; Gorel et al. 2021; Schulz et al. 2022).

**Fig. 1:**
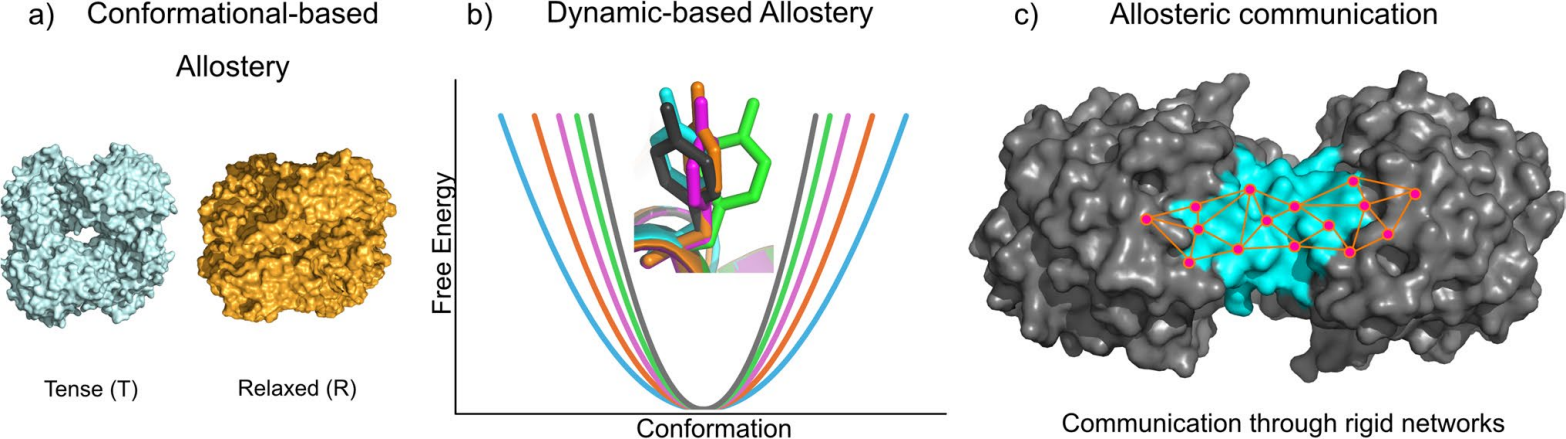
Models of allostery. **a** Conformation-based model of allostery which is best represented by the MWC and KNF models. **b** Dynamic-based model of allostery where internal motions of the protein follow the energy landscape. **c** Communication of allosteric effects are usually driven through rigid networks. Adapted from Wu et al. (2024)

In parallel, advances in spectroscopic techniques, such as solution NMR, have unveiled a more nuanced picture of allosteric proteins, revealing their dynamic nature (Cooper and Dryden 1984; Tzeng and Kalodimos 2011, 2012; Lechtenberg et al. 2012; McLeish et al. 2015; Guo and Zhou 2016). This has led to a broader definition of allostery, encompassing the propagation of energy sometimes through rigid pathways, even in simple proteins, forming what are now recognized as “allosteric networks.” Understanding these networks is crucial for elucidating how proteins function and regulate biological processes. While traditional static crystallography methods are invaluable, they can on rare occasions capture population shifts as described by Shibayama and colleagues, using a crystal form of hemoglobin with multiple conformations simultaneously present (Shibayama et al. 2017). However, the technique often falls short in capturing the dynamic nature of allosteric events, such as conformational changes and ligand binding (Fig. 1). Controlling a variable such as temperature can reveal states that can otherwise be hidden at cryogenic temperatures (Fraser et al. 2009; Keedy et al. 2014, 2015b). Time-resolved crystallography addresses this gap by allowing researchers to visualize allosteric transitions at the atomic level in real time, thus revolutionizing our understanding of these processes (Knapp et al. 2006; Shi and Kay 2014; Mehrabi et al. 2019b).

Cross pollination of kinetics and spectroscopic methods into more structure focused techniques, coupled with the development of advanced in silico methods, has significantly enhanced our ability to explore proteins and understand their intricate structure–function-dynamics relationships (Szymczyna et al. 2009; Kim et al. 2017; Tararina et al. 2018; Mehrabi et al. 2019a). Moreover, advances in data collection, detectors, and new crystallographic techniques, which can observe the protein of interest out of equilibrium, have emerged, providing even greater insights and improving our capacity to investigate these complex and fascinating processes(Chapman 2019). These advances collectively push the boundaries of our knowledge, enabling a deeper and more comprehensive understanding of allosteric regulation.

Combining techniques which attempt to control multiple variables that push the system out of equilibrium and along its reaction coordinate is where multi-dimensional crystallography comes in, which encompasses variations in time, temperature, or even humidity, allowing researchers to capture snapshots of protein dynamics near physiological conditions. In this review, we explore how these techniques, particularly time-resolved crystallography and temperature-dependent crystallography, have shed new insights on our understanding of allosteric regulation. Furthermore, we examine the synergistic combination of time and temperature with multi-dimensional crystallography, to provide comprehensive insights into allosteric processes.

### X-ray crystallography and temperature-dependent allostery

In the context of allostery, X-ray crystallography has been one of the leading methods used for demonstrating the structural basis for induced conformational changes upon ligand binding. Historically, during the first decades since the advent of protein crystallography, the majority of diffraction data from protein crystals were collected at ambient temperature(Kendrew et al. 1958; Perutz et al. 1960; Blake et al. 1967). Although there were a few cryo-cooling experiments performed in the 1960s to demonstrate feasibility (Haas 2020), it was not until the 1990s that vitrifying protein crystals prior to placing them in the X-ray beam became a ubiquitous practice. Cryocrystallography is a technique where protein crystals are flash-cooled to cryogenic temperatures (typically around 100 K) to slow radiation damage during X-ray exposure and this improve the overall resolution of the final diffraction dataset (Pflugrath 2015). While cryocrystallography was originally developed to preserve the integrity of the crystal, it also “freezes” the protein in a specific conformation (or set of closely related conformations), allowing the capture of high-resolution structures of highly populated long-lived intermediates (Garman and Schneider 1997). While the advantages of cryo-cooling crystals are numerous, and it is still the workhorse technique for collecting single crystal data, this technique can reduce populations of some conformational states that would be visible were data collected at ambient or higher temperatures. During the cooling process the protein can be pushed into an energy minimum in which it cannot sample certain conformational states, and therefore the resulting ensemble average structure obtained by crystallography does not provide a complete picture of its equilibrium dynamic behavior (Fraser et al. 2011; Keedy et al. 2014). However, with recent advances in detectors, new 3rd/4th gen synchrotrons, and the advent of XFELs, collecting data at room temperature is becoming more routine again(Schlichting 2015; Chapman 2019; Pearson and Mehrabi 2020).

**Fig. 2:**
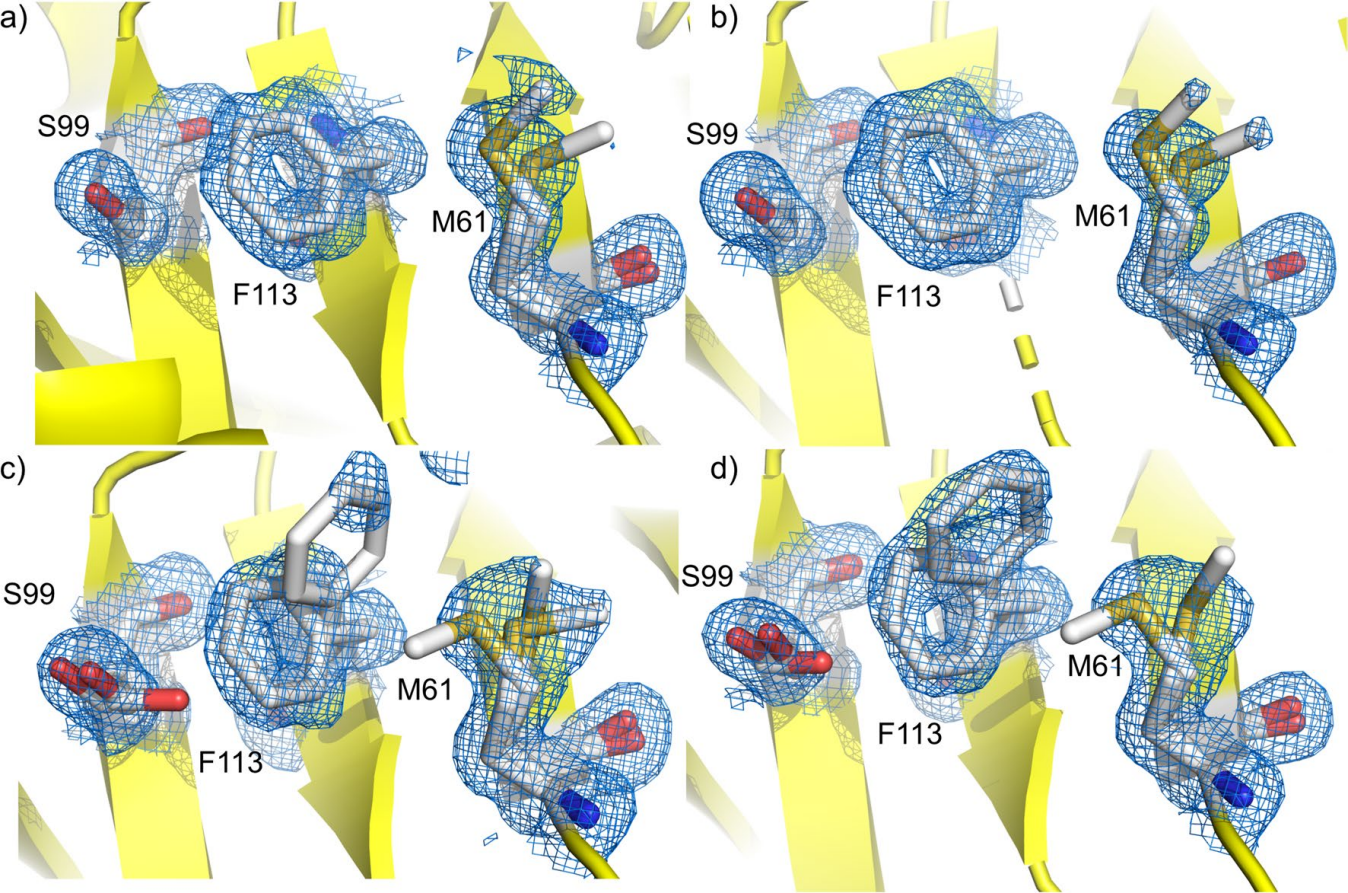
Increased conformational heterogeneity across temperature. **a** 100 K, **b** 180 K, **c** 260 K and **d** 300 K. The cyan 2mFo-DFc electron density is modelled at 1.0 σ. Adapted from Keedy et al. (2015b)

An extension of collecting data from protein crystals at ambient temperatures is temperature-dependent X-ray crystallography. This technique involves studying structural changes in proteins while varying temperature over several tens of degrees in order to understand how temperature affects protein dynamics and stability. By changing the temperature during data collection, it is possible to observe how temperature influences both protein conformation and allosteric regulation (Weik and Colletier 2010; Keedy 2019; Knight et al. 2023). This approach provides insights into the thermodynamic and kinetic aspects of allosteric transitions, allowing for the observation of temperature-dependent structural changes (Fischer 2021). Over the past decade, seminal work has shown that while cryogenic temperatures provide high-resolution static structures, they often miss dynamic conformational states critical for protein function. At ambient temperatures, crystal structures have revealed previously hidden minor conformations and interconverting substates, and demonstrated how these dynamic states contribute to enzymatic activity (Fraser et al. 2011; Keedy et al. 2015b, a; Skaist Mehlman et al. 2023) (Fig. 2).

For instance, a study on the signaling switch protein H-Ras compared structures at both cryogenic and room temperatures to investigate temperature-dependent conformational changes. The results revealed an allosteric network, previously detected by NMR, that was not observable in the electron-density maps of vitrified samples at 100 K, highlighting the importance of temperature in modulating enzyme activity (Fraser et al. 2011). Similarly, with protein tyrosine phosphatase 1B (PTP1B), data collected over a wide range of temperatures revealed new allosteric networks converging on the active site (Keedy et al. 2018). Additionally, serial crystallography, and by extension time-resolved serial crystallography, is routinely performed at room temperature. This technique will be briefly discussed in the next section.

Despite the challenges associated with data collection at higher temperatures, such as increased rates of radiation damage, issues with sample dehydration, and increased diffuse scatter, careful consideration of the experimental parameters can result in much richer data (Thorne 2023). Overcoming these obstacles allows researchers to gain a more comprehensive understanding of protein dynamics and allosteric regulation. Expanding on these findings, temperature-dependent X-ray crystallography helps bridge a gap between static and dynamic structural data but also offers a more realistic view of protein behavior under physiological conditions.

### Time-resolved crystallography: probing allosteric dynamics

Time-resolved crystallography (TRX) is a fast-developing field that enables capturing snapshots of a protein’s structure, while it is traversing its reaction coordinate, over a very wide range of time points. Originally, TRX techniques used Laue diffraction, which uses polychromatic (pink) X-rays to capture events as fast as 100 ps using light driven pump-probe methods(Ren et al. 1999; Schotte et al. 2004; Meents et al. 2017). Recent advancements in X-ray free-electron lasers (XFELs), along with the advent of serial crystallography, have significantly enhanced the temporal resolution of TRX down to femtoseconds (Chapman 2019; Pearson and Mehrabi 2020). The ultra-short femtosecond pulses provided by XFELs allow visualization of fast dynamic processes and capturing of transient states in real time with near zero radiation damage (Nass Kovacs et al. 2019; Woodhouse et al. 2020). Many of the techniques and technologies developed by the XFEL community have been transferred to the synchrotron community, allowing time-resolved serial crystallography at microfocus beamlines to capture interesting biochemical processes, albeit at slower timepoints (µs – s) (Roedig et al. 2016; Beyerlein et al. 2017; Mehrabi et al. 2019b; Pearson and Mehrabi 2020). Unlike traditional X-ray crystallography, which provides ensemble averaged “static” structures, TRX can reveal the sequence of structural changes that occur during biochemical reactions, with the study of fast dynamic processes such as charge transfer, bond breakage and formation, and vibration transfer possible at XFELs, and slower processes such as enzyme catalysis, ligand binding, and allosteric transitions, also accessible at synchrotrons (Chapman 2019; Pearson and Mehrabi 2020).

One of the advantages of TRX is that it allows researchers to observe structural transitions in real time, with the system pushed out of its ground state. This provides a picture of both short and long-lived intermediates, and the conformational transition of populations between these states. By initiating a reaction in the crystal and capturing structural snapshots at various times, time-resolved crystallography (TRX) can provide extensive insights into catalysis and the structural characteristics of allostery (Pande et al. 2016; Mehrabi et al. 2019b; Woodhouse et al. 2020; Nass et al. 2020). Allosteric communication can involve complex conformational changes and very subtle, almost structurally hidden, transitions that require careful interrogation of the data to detect and that are sometimes difficult to capture (Tzeng and Kalodimos 2011; Mehrabi et al. 2019a; Wu et al. 2024). The advent of serial crystallography has seen a new resurgence of the field since the early work with Laue beamlines in the 1990s and 2000s, with numerous studies demonstrating the effectiveness of time-resolved studies in examining protein dynamics such as isomerization, binding, and turnover, even for non-state-reversible systems (Milani et al. 2008; Barends et al. 2015; Pande et al. 2016; Mehrabi et al. 2019b; Nass Kovacs et al. 2019). While there have been several papers showing various forms of protein dynamics, two compelling examples of using TRX to capture both cooperative and allosteric behavior are in heme proteins and in fluoroacetate dehalogenase (FAcD) (Knapp et al. 2006; Mehrabi et al. 2019b). For instance, a study of *Scapharca* dimeric hemoglobin (HbI) revealed a two-phase structural transition from the R state to the T state following ligand photodissociation. An early intermediate formed within 5 ns, characterized by heme center buckling, movement of neighboring side chains and helices, and disruption of R-state water molecules. This was followed by an allosteric phase lasting from 1 to 80 µs, during which cooperative structural changes propagated, culminating in the formation of the tertiary T state. The study also showed that the transition back to the R state upon ligand rebinding is significantly slower, suggesting a stable T state even in singly liganded species (Knapp et al. 2006).

**Fig. 3:**
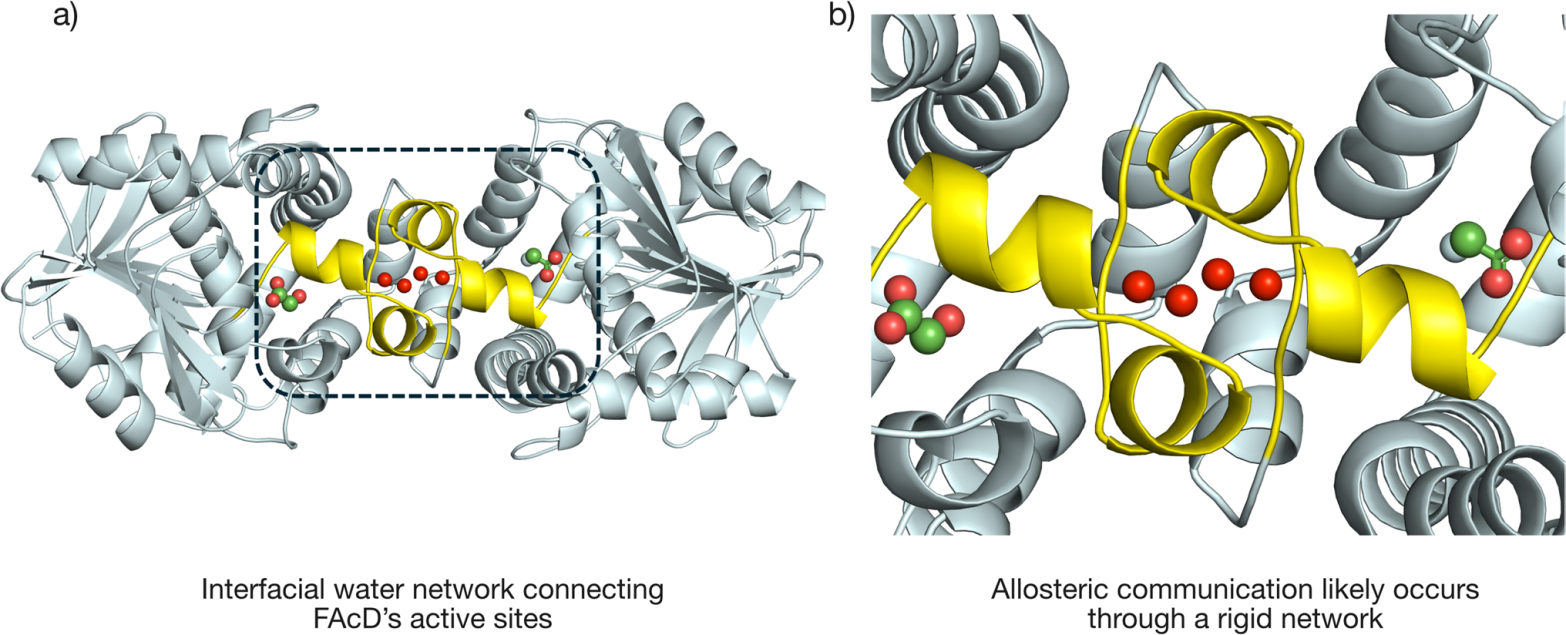
Proposed allosteric water chain linking both active sites. **a** Interfacial waters connecting the subunits of FAcD. **b** The two subunits are connected via an allosterically coupled rigid network, which is a molecular framework of atoms and connecting covalent and hydrogen bonding interactions

A more recent study by explored the structural dynamics associated with the full-turnover of enzymatic catalysis using time-resolved serial synchrotron crystallography (TR-SSX) to capture key states of the homodimeric enzyme fluoroacetate dehalogenase (FAcD) from *Rhodopseudomonas palustris* (Mehrabi et al. 2019b). This enzyme is capable of breaking carbon–fluorine bonds and is of potential interest for bioremediation purposes (Chan et al. 2011; Kim et al. 2017). Eighteen structures from 0 to ~ 30 s revealed sequential substrate binding, covalent intermediate formation, and product release, in each of the individual subunits, highlighting FAcD’s half-of-the-sites reactivity, meaning that only one active site is reactive at a time, a common feature in allosteric enzymes. It was postulated that structural waters at the interface between the enzyme’s subunits play a crucial role in facilitating allosteric communication as changes in the number and positioning of these water molecules accompany the different catalytic steps. Importantly, many of the enzyme’s dynamic features seemed to align at key steps along the catalytic cycle. These findings provided insights into the enzyme’s allosteric regulation, emphasizing the importance of structural waters and molecular dynamics in enzymatic function (Mehrabi et al. 2019b) (Fig. 3).

### Towards multi-dimensional data

Combining techniques from time-resolved crystallography and temperature-dependent crystallography offers a powerful approach for uncovering the dynamic landscape of allosteric mechanisms. By simultaneously capturing the temporal and thermal fluctuations within protein structures, we can begin to gain a more comprehensive understanding of how proteins transition between different functional states. For instance, TRX allows experimental tracking of conformational changes over a series of time points following ligand binding or other perturbations. Meanwhile, modulating temperature provides insights into how temperature variations influence protein flexibility, stability, and interactions. The integration of these techniques can reveal otherwise low populated intermediates and subtle conformational changes that might be missed in a single temperature experiment. For example, the first proof-of-principle study on photoactive yellow protein (PYP) collected a time and temperature series from large single crystals using pink beam time-resolved diffraction techniques (Schmidt et al. 2010). This carefully designed study showed that it is, in principle, possible to obtain relaxation times from the crystallographic data and therefore obtain a kinetic mechanism directly from the structural analysis (Schmidt et al. 2013). Emerging methods are currently being developed to take advantage of the additional non-state-reversible systems that serial time-resolved crystallography makes accessible. This includes keeping radiation damage as low as reasonably possible, as well as making it simpler to probe irreversible non-light activable systems as a function of temperature (Schulz et al. 2024).

A study by Wolff et al. (2023) demonstrated a novel method using temperature jumps via an IR laser to perturb the solvent bath around the protein and subsequently observing the coupling of the thermal fluctuations of the surrounding solvent to the dynamics of the protein at various timepoints at an XFEL (Wolff et al. 2023) (Fig. 3). Another example, which took a different approach, is the development of an environmental chamber for fixed-target serial crystallography, which can control both temperature and humidity around the sample with a high degree of precision. Being able to vary the temperature and humidity and keep a specific set of parameters fixed for the sample during a time-resolved measurement allows for a great range of experimental flexibility (Schulz et al. 2024).

## Challenges and future directions

The collection of crystallographic data beyond simple “static” structures can become significantly more complex. Each additional dimension, from time to temperature, adds a layer of complexity that needs to be carefully controlled, to ensure consistent and accurate data collection across different conditions. Careless mistakes or poor experimental design can result in the introduction of artifacts—e.g., from radiation damage. Care is also required in the structural modelling of low-populated states, in order to avoid over-fitting or over-interpreting electron density features which may not be biologically relevant, potentially leading to misleading conclusions about allosteric mechanisms. The data provided by TRX are currently of insufficient quality to enable the refinement of models against them. This is due to the fact that there are multiple states that exist at any one time, whereas there is usually only a single model that is refined against it. The number of parameters required to deliver such a full model of the entire dynamic population is not yet refinable, because the data are currently not strong enough. However, time-resolved data is highly effective for hypothesis testing and for integrating spectroscopic, computational, and high-resolution models along a reaction coordinate.

To confirm and validate biological mechanisms, it is therefore crucial to employ complementary biophysical techniques. While advanced crystallographic methods are beginning to provide new information, delving into understanding allostery requires a multidisciplinary approach. For example, spectroscopic techniques such as electronic and vibrational spectroscopy and NMR spectroscopy are extremely useful as they provide information about reaction kinetics, relaxation rates, dipolar couplings, and other insights into protein dynamic behavior. Spectroscopic data complement crystallographic findings by providing a more continuous view of structural transitions and the timescales over which they occur. Molecular dynamics (MD) simulations and other in silico methods provide information inaccessible experimentally, either due to sparsely populated states or experimentally uncapturable timescales. Likewise, other computational methods for predicting allosteric pathways and temperature-dependent allosteric transmissions, such as rigidity transmission analysis (RTA) which uses concepts from combinatorial rigidity theory to map out transmission between rigid bodies of the protein, can be used to map out potential allosteric pathways (Sljoka 2012; Kim et al. 2017; Mehrabi et al. 2019a).

## Conclusion

By structurally visualizing these complex behaviors, researchers can build more accurate models of protein dynamics, enhancing our understanding of biological function and guiding the design of allosteric modulators with therapeutic potential. The holistic perspective gained from integrating time-resolved and variable temperature crystallography is crucial to model protein behavior. It bridges the gap between “static” structural snapshots and dynamic processes, offering a richer, more detailed picture of protein function. These multi-dimensional approaches, while still in their infancy, have the potential to provide a more comprehensive view of the structural dynamics and kinetic mechanisms involved in protein function and, in particular, the molecular basis of allosteric communication and regulation.

## Data Availability

No datasets were generated or analysed during the current study.
